# Genetic deficiency of protein inhibitor of activated STAT3 suppresses experimental abdominal aortic aneurysms

**DOI:** 10.3389/fcvm.2023.1092555

**Published:** 2023-03-15

**Authors:** Weilai Fu, Haole Liu, Panpan Wei, Congcong Xia, Qingqing Yu, Kangli Tian, Yankui Li, Enqi Liu, Baohui Xu, Masaaki Miyata, Rong Wang, Sihai Zhao

**Affiliations:** ^1^ Institute of Cardiovascular Science, Translational Medicine Institute, Xi'an Jiaotong University Health Science Center, Xi’an, China; ^2^ Department of Vascular Surgery, The Second Hospital of Tianjin Medical University, Tianjin, China; ^3^ Laboratory Animal Center, Xi'an Jiaotong University Health Science Center, Xi’an, China; ^4^ Department of Surgery, Stanford University School of Medicine, Stanford, CA, United States; ^5^ School of Health Science, Faculty of Medicine, Kagoshima University, Kagoshima, Japan

**Keywords:** abdominal aortic aneurysm, protein inhibitor of activated STAT3, inflammation, macrophage, animal model

## Abstract

**Aim:**

Signal transducer and activator of transcription (STAT) signaling is critical for the pathogenesis of abdominal aortic aneurysms (AAAs). Though protein inhibitor of activated STAT3 (PIAS3) negatively modulates STAT3 activity, but its role in AAA disease remains undefined.

**Method:**

AAAs were induced in PIAS3 deficient (PIAS3^−/−^) and wild type (PIAS3^+/+^) male mice *via* transient intra-aortic elastase infusion. AAAs were assessed by *in situ* measurements of infrarenal aortic external diameters prior to (day 0) and 14 days after elastase infusion. Characteristic aneurysmal pathologies were evaluated by histopathology.

**Results:**

Fourteen days following elastase infusion, aneurysmal aortic diameter was reduced by an approximately 50% in PIAS3^−/−^ as compared to PIAS3^+/+^ mice. On histological analyses, PIAS3^−/−^ mice showed less medial elastin degradation (media score: 2.5) and smooth muscle cell loss (media score: 3.0) than those in PIAS3^+/+^ mice (media score: 4 for both elastin and SMC destruction). Aortic wall leukocyte accumulation including macrophages, CD4^+^ T cells, CD8^+^ T cells and B cells as well as mural neovessel formation were significantly reduced in PIAS3^−/−^ as compared to PIAS3^+/+^ mice. Additionally, PIAS3 deficiency also downregulated the expression levels of matrix metalloproteinases 2 and 9 by 61% and 70%, respectively, in aneurysmal lesion.

**Conclusion:**

PIAS3 deficiency ameliorated experimental AAAs in conjunction with reduced medial elastin degradation and smooth muscle cell depletion, mural leukocyte accumulation and angiogenesis.

## Introduction

Abdominal aortic aneurysm (AAA) is a lethal degenerative disease that is prevalent in older smoker men (1, 2). Inflammation is one of well-established pathophysiological mechanisms in the genesis of AAAs. For example, the circulating levels of inflammatory cytokines such as interleukin (IL)-6, IL-1β, tumor necrosis factor (TNF)-*α* and other mediators were elevated in patients with AAAs and may mediate AAA formation and progression (3–5). Inhibition of inflammatory cytokines effectively attenuated experimental AAA formation (6–8).

Janus kinase (JAK)/signal transducer and activator of transcription (STAT) signaling critically regulates inflammatory responses (9, 10). It is involved in cytokine production, immune cell recruitment, and initiation of adaptive responses (9, 11). Four JAKs (JAK1-JAK3 and tyrosine kinase 2) and seven STATs (STAT1-STAT4, STAT5a, STAT5b, and STAT6) are present in mammalian cells (12). JAK2/STAT3 signaling modulates cell proliferation as well as cell survival. Additionally, enhanced JAK2/STAT3 signaling activity has been reported in clinical AAA specimens (13).

Excessively activating JAK/STAT signaling leads to dysregulated immune responses and thus tissue damage. Several endogenous inhibitory proteins, such as protein inhibitor of activated STAT (PIAS) and suppressor of cytokine signaling (SOCS), are evolved to limit overwhelming inflammation due to augmented JAK/STAT activity. We previously showed that PIAS3 was reversely associated with atherosclerotic progression and inhibited inflammatory responses and smooth muscle cell (SMC) proliferation (14). AAA as a chronic inflammatory disease may share some common pathogenic pathways with atherosclerosis. The role PIAS3 plays in AAAs has not been clarified. Therefore, the present study was to investigate the influence of PIAS3 deficiency on experimental AAAs in the elastase-induced AAA model.

## Materials and methods

### Animals

**Figure 1:**
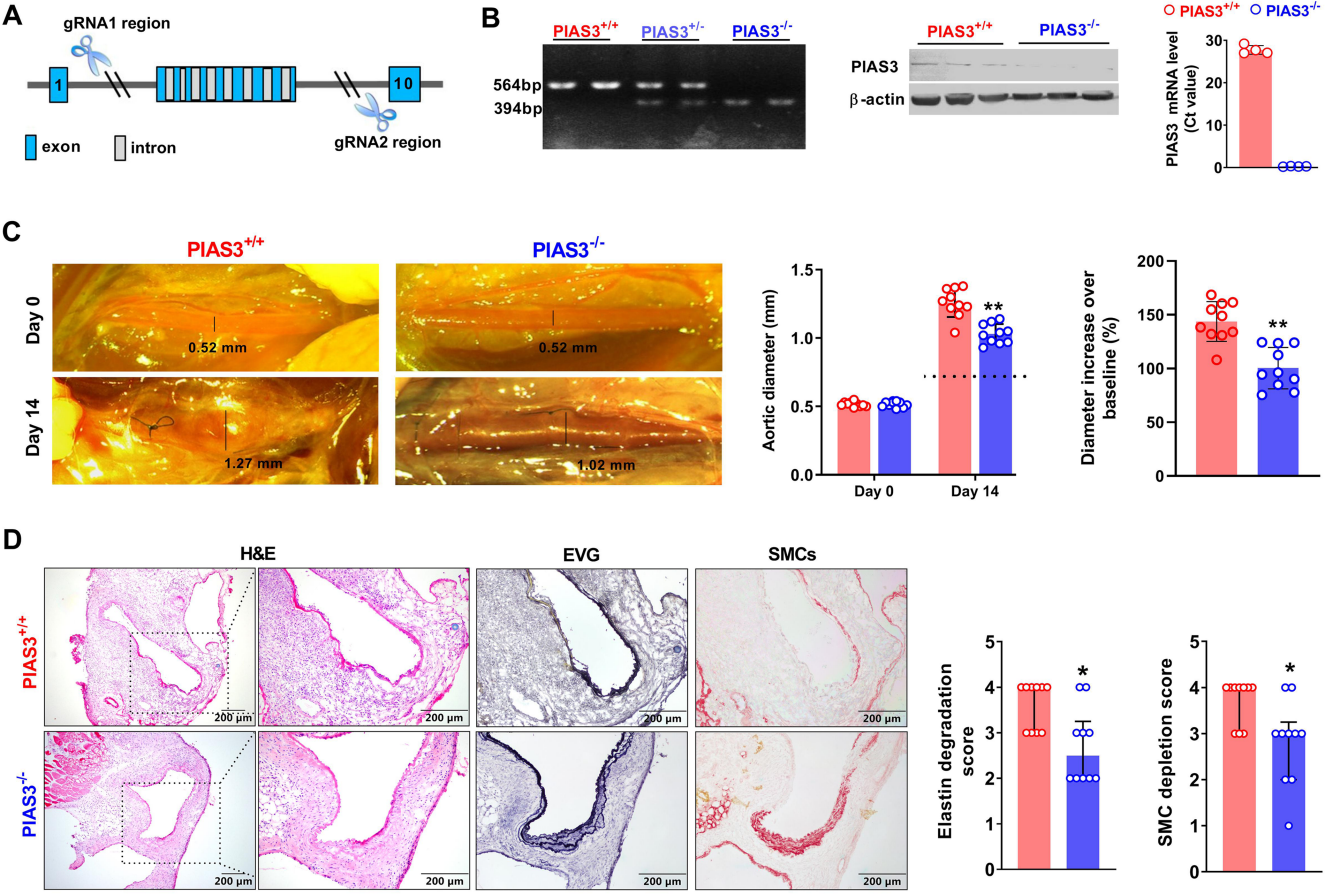
Genetic deficiency of PIAS3 suppresses experimental AAAs. (**A**): Strategy for generating PIAS3 deficient (PIAS3^−/−^) mice by targeting exons 2-9 *via* the CRISPR/Cas9 technique. (**B**): Characterization of PIAS3^−/−^ mice by PCR genotyping, qRT-PCR and Western blotting analyses. **(C**): Representative aortic photographs and quantification of aortic diameters on day 0 and day 14 after elastase infusion. Dotted line indicates average aortic diameter after PBS infusion (approximately 0.8 mm). (**D**): Representative images of H&E, EVG and SMC *α*-actin staining as well as the semi-quantification (media and interquartile) of aneurysmal medial elastin and SMC destruction. Two-way ANOVA followed two group comparison (**C**) and non-parametric Mann-Whitney test (**D**). *N* = 10 for each group, *p < 0.05 and **p < 0.01 as compared to PIAS3^+/+^ mice.

PIAS3 gene (NM_001165949) is located on mouse chromosome 3 with fourteen exons (15). PIAS3 deficient (PIAS3^−/−^) mice on C57BL/6 genetic background were created in Cyagen Biosciences (Suzhou) Inc (Taicang, Jiangsu, China) using the CRISPR/CAS9 technique. Briefly, the exons of 2–9 were selected as target sites, and two gRNAs with Cas9 mRNA were microinjected into zygotes for PIAS3 deficient mouse generation (Figure 1A). F0 founders were identified *via* PCR genotyping followed by DNA sequencing. Homozygotes (PIAS3^−/−^) and wild type (PIAS3^+/+^) littermates were screened and used for all experiments. The use and care of animals as well as all experimental procedures were reviewed and approved by the Laboratory Animal Administration Committee of Xi'an Jiaotong University (No. 2019–1178). All information on animals and related reagents were detailed in Supplementary Table S1.

### Phenotyping of PIAS3 deficient mice

For PCR screening, tail tip samples were collected from <3 weeks old mice and genomic DNA was extracted by proteinase K digestion. Genotyping PCR primers were AAACAAGACTAAAGGAGTATGGGC (sense 1), TAGAGGAAGGGGAAGGGAACTAAG (antisense 1) and CTCAGACACTCGGAAACTCATC (antisense 2). For quantitative reverse transcription PCR (qRT-PCR) and Western blotting analyses, total RNA and proteins were extracted from aortas. The PIAS3 primers for qRT-PCR analysis were sense: ACCAAGAATGGAGCTGAGCC (sense) and TCTGGATTCCGGATCCCCTT (antisense). Antibodies against PIAS3 and *β*-actin were obtained from Cell Signaling Technology (Danvers, MA, United States) and TransGen Biotech Co., Ltd (Beijing, China), respectively.

### Experimental AAA modeling

Experimental AAAs were induced in PIAS3^−/−^ and PIAS3^+/+^ mice by transient luminal infusion of porcine pancreatic elastase (PPE, 1.5 units/mL in phosphate-buffered saline) in controlled infrarenal aortic segment as previously reported (16, 17). Briefly, 9–12 weeks old male mice were anesthetized by 2% isoflurane inhalation, and infrarenal aorta was exposed *via* a laparotomy. Infrarenal aorta was then infused with PPE solution *via* an aortotomy for 5 min under constant pressure as previously described (18). After the surgery, all mice were maintained in individual cages with free access to chow diet and water.

### Measurement of abdominal aortic diameters

Prior to PPE infusion, infrarenal aorta was photographed with a digital camera. The diameter was measured using Images Plus3.0 ML (Motic Electric Group Co., Ltd, Xiamen, Fujian, China) and served the baseline level. On day 14 after PPE infusion, infrarenal aortic diameter for each mouse was measured by the same procedure prior to sacrifice and aorta was then harvested. A mouse with a more than 50% aortic dilation over the baseline was considered aneurysmal (18).

### Analysis of medial elastin and SMCs

Mice were euthanized by carbon dioxide inhalation. PPE-infused aortic segment was collected, embedded in optimal cutting temperature media, and sectioned (6 μm). Frozen sections were stained with hematoxylin and eosin (H & E) and Elastic van Gieson (EVG) stains, respectively, for the assessment of general morphology and elastin integrity. Aortic media elastin degradation was graded as I (mild) to IV (severe) on EVG-stained sections as previously reported (16–20). To evaluate SMC depletion, acetone-fixed frozen sections were stained with a goat anti-SMC *α*-actin antibody followed a standard biotin-streptavidin peroxidase procedure. Biotinylated donkey anti-goat IgG antibody and streptavidin-peroxidase conjugate were obtained from the Jackson ImmunoResearch Laboratories Inc., West Glove, PA, United States). AEC substrate kit for color development was purchased from Vector Laboratories Inc., Burlingame, CA, United States. Aortic medial SMC depletion was graded on a scale of I (mild) to IV (severe) as reported previously (20).

### Analysis of aortic leukocytes

Acetone-fixed aortic frozen sections were stained with monoclonal antibodies against CD68 (macrophages), CD4^+^ T cells, CD8^+^ T cells and B cells (B220) (18). Sections were sequentially incubated with biotinylated goat anti-rat antibody (Vector Laboratories Inc), PBS, streptavidin-peroxidase conjugate (Jackson ImmunoResearch Laboratories Inc), PBS, peroxidase substrate AEC (Vector Laboratories Inc), counterstained with hematoxylin, mounted and cover-slipped. Aortic mural macrophage accumulation was graded on a scale of I (mild) to IV (severe) (20). CD4^+^ T cells, CD8^+^ T cells and B cells were quantitated as positively stained cells per aortic cross section (ACS) (20).

### Immunostaining of aneurysmal matrix metalloproteinases

Matrix metalloproteinases (MMPs), particularly MMP2 and MMP9, contribute to the pathogenesis of AAAs. MMP2 and MMP9 were assessed by immunostaining using goat anti-mouse polyclonal antibodies against MMP2 (AF1488) and MMP9 (AF909) (R & D Systems, Minneapolis, MN, United States) (Cite your JIR article). The expression levels were quantitated as the positively stained area per ACS using the image analysis software (WinRoof 6.5, Mitani Co. Ltd., Tokyo, Japan).

### Analysis of mural angiogenesis

Angiogenesis was analyzed by immunostaining with rat anti-mouse CD31 monoclonal antibody (Clone 390, Biolegend Inc, San Diego, CA, United States) previously described. The neovessels number were counted under the microscope and reported as CD31-positive vessels per ACS (20, 21).

### Statistical analysis

Prism 9.0 software was used for all statistical analyses. Continuous variables were expressed as mean and standard deviation (normal distribution) or media with interquartile (not normal distribution). Student's *t* and nonparametric Mann-Whitney tests were used for normally and nonnormally distributed data, respectively. Two-way ANOVA followed by Sidak's multiple comparisons test was used for testing statistical difference for aortic diameters among groups. Statistical significance level was set at *p* < 0.05.

## Results

### Generation of PIAS3 knockout mice

As illustrated in Figures 1A,B, PIAS3 deficient mice were successfully generated by CRISPR/Cas9 techniques. PCR genotyping demonstrated the homozygotes of PIAS3 deficiency. qRT-PCR and Western blotting analysis further confirmed the deficiency of PIAS3 at mRNA and protein levels in PIAS3^−/−^ as compared to PIAS3^+/+^ mice.

### PIAS3 deficiency suppresses PPE-induced aortic dilation in mice

Intra-infrarenal aortic PPE infusion was conducted to induce AAAs in PIAS3^+/+^ and PIAS3^−/−^ mice. Experimental AAAs were successfully induced in PIAS3^+/+^ mice (Figure 1C). PIAS3 deficiency significantly inhibited aortic expansion as compared to PIAS3^+/+^ mice. Aortic diameter on day 14 after PPE infusion were 1.03 ± 0.07 mm and 1.26 ± 0.10 mm for PIAS3^−/−^ and PIAS3^+/+^ mice, respectively (Figure 1C). After subtracting an average aortic dilation caused by PBS infusion (approximately 0.8 mm), PIAS3 deficiency reduced PPE-induced aortic expansion by approximately 50% (Figure 1C, middle panel). The diameter increase over the baseline was significantly less in PIAS3^−/−^ than that in PIAS3^+/+^ mice (Figure 1C, right panel).

### PIAS3 deficiency ameliorates medial elastin degradation and SMC depletion

Histological analysis was performed in aneurysmal aortas. In H&E staining, PPE infusion induced a remarkable aortic dilation, predominant inflammatory cell infiltration, medial elastin degradation and SMC loss (Figure 1D, left panel). However, PIAS3^−/−^ mice were protective against the destruction of medial elastin and SMCs. In comparison with PIAS3^+/+^ mice, the integrity of medial elastin was relatively preserved in PIAS3^−/−^ mice, with significantly reduced elastin degradation score (Figure 1D, middle panel). Similar was true for medial SMCs (Figure 1D, right panel).

### PIAS3 deficiency inhibits aortic leukocyte accumulation

**Figure 2:**
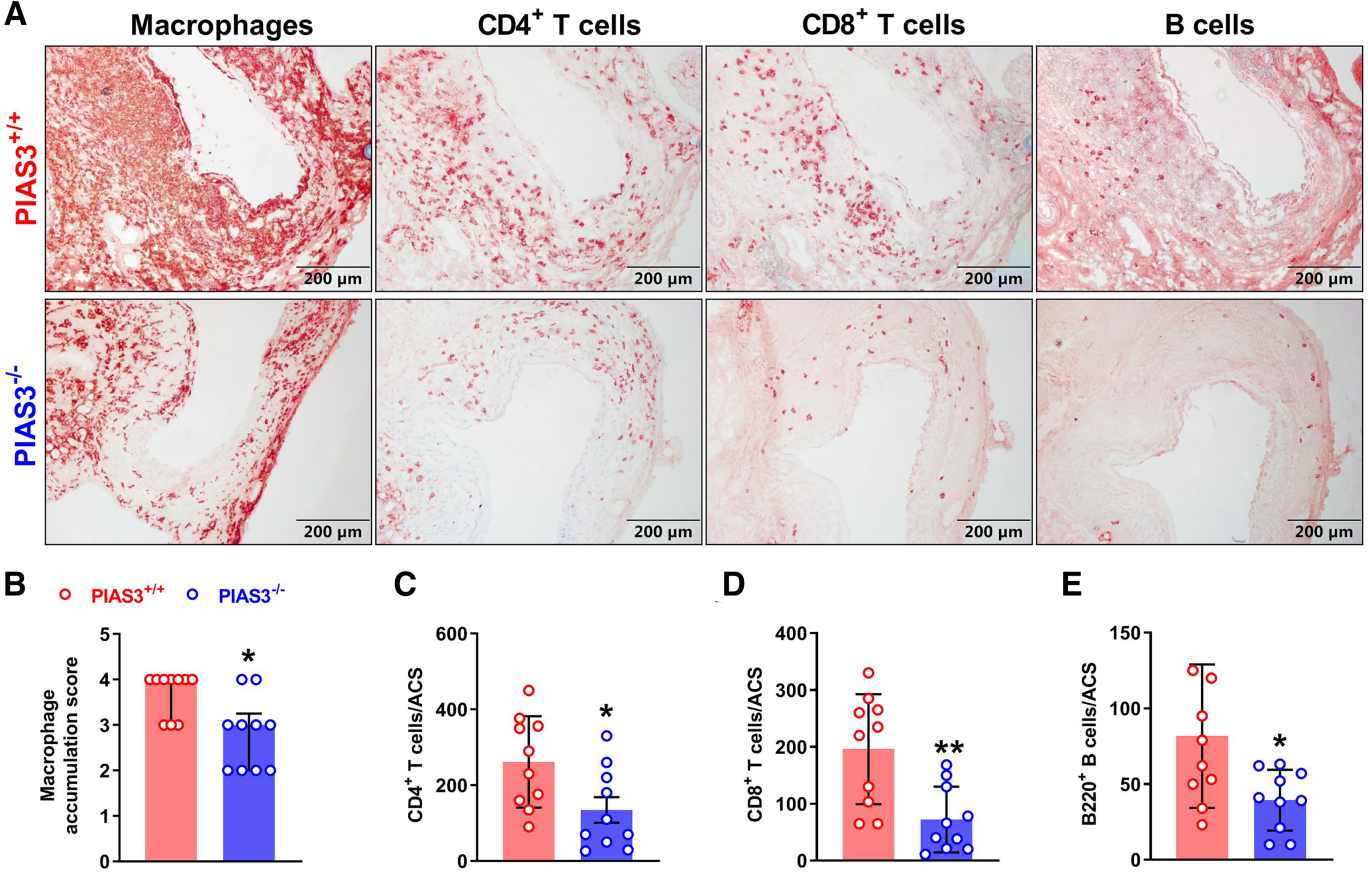
PIAS3 deficiency inhibits aneurysmal aortic leukocyte accumulation. (**A**): Representative images of immunohistochemical staining showing aneurysmal aortic leukocyte accumulation (macrophages, T cells and B cells). (**B-E**): Semi-quantification of aneurysmal aortic leukocyte accumulation. Aortic mural macrophage accumulation was scored from I to IV, while aortic CD4^+^ T cells, CD8^+^ T cells and B cells were quantified as positively stained cells number per aortic cross-section (ACS). Data are shown as media and interquartile (**B**) and mean and standard deviation (**C–E**). Non-parametric Mann-Whitney test (**B**) and Student's *t* test (**C–E**). *N* = 10 for each group, *p < 0.05 and **p < 0.01 as compared to wild type (PIAS3^+/+^) mice.

In aortic immunostaining, the score for the accumulation of macrophages, identified by CD68-positve cells, was significantly lower in PIAS3^−/−^ than that in PIAS3^+/+^ mice (Figures 2A,B). Similarly, PIAS3 deficiency reduced the accumulation of CD4^+^ T cells, CD8^+^ T cells and B cells by approximately 50% (Figure 2).

### PIAS3 deficiency reduces aortic MMP2 and MMP9 expression

**Figure 3:**
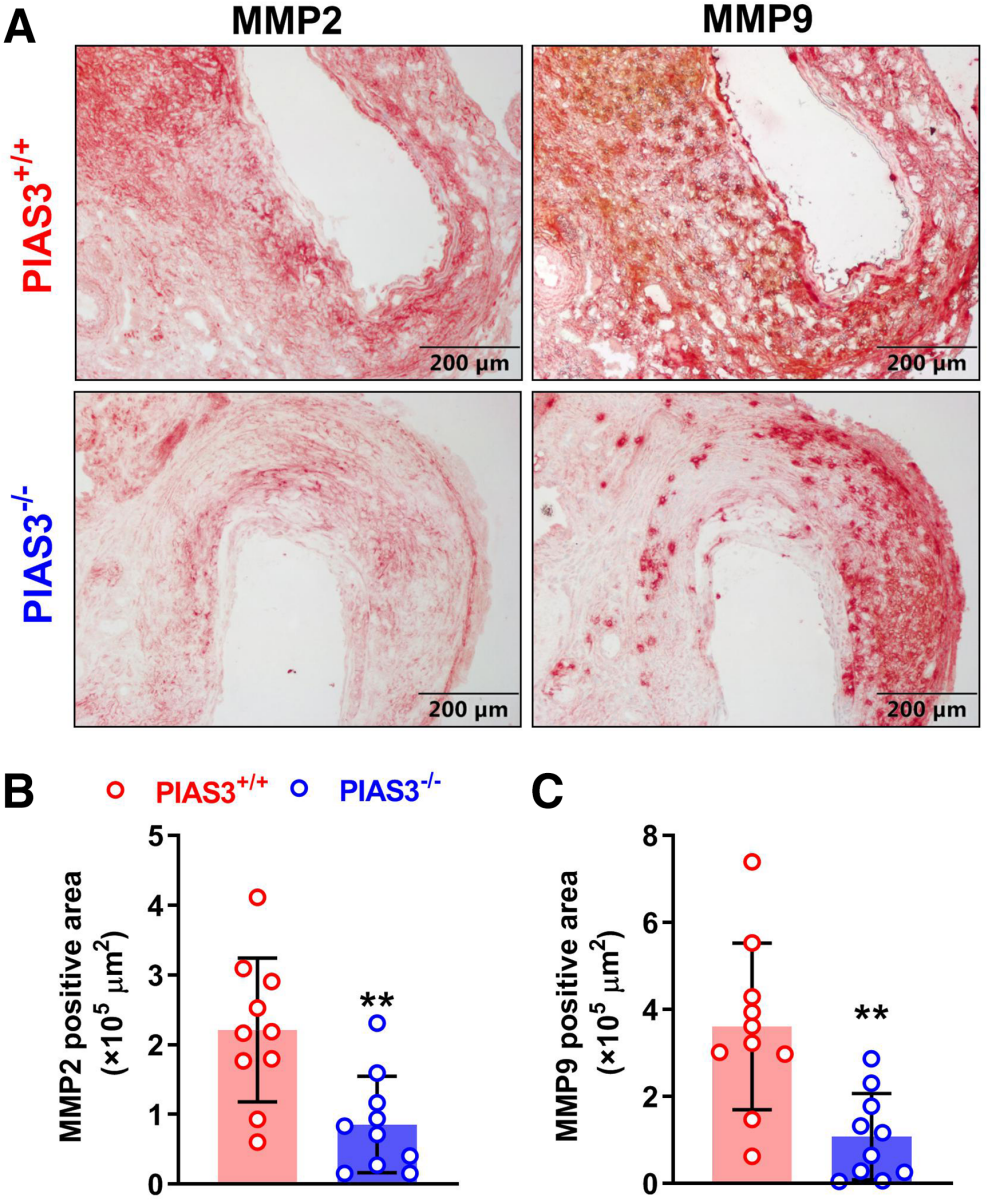
PIAS3 deficiency decreases the expression levels of matrix metalloproteinases 2 and 9. (**A**): Representative immunohistochemical staining images of MMP2 and MMP9 in aneurysmal aortas from mild type (PIAS3^+/+^) and PIAS3 deficient (PIAS3^−/−^) mice. (**B**): Semi-quantification of MMP2 and MMP9 expression levels in aneurysmal lesions quantitated as the positively stained area per aortic cross-section. *N* = 10 for each group, *p < 0.05 and **p < 0.01 as compared to PIAS3^+/+^ mice.

MMP2 and MMP9 are contributors of PPE-induced AAAs (17, 22, 23). The levels of aortic MMP2 and MMP 9 were diminished in PIAS3^−/−^ as compared to PIAS3^+/+^ mice (Figure 3A). In semi-quantitative analysis, individual MMP positively stained areas were reduced by approximately 61% and 70% for MMP2 and MMP9, respectively, in PIAS3^−/−^ as compared to PIAS3^+/+^ mice (Figures 3B,C).

### PIAS3 deficiency suppresses mural angiogenesis

**Figure 4:**
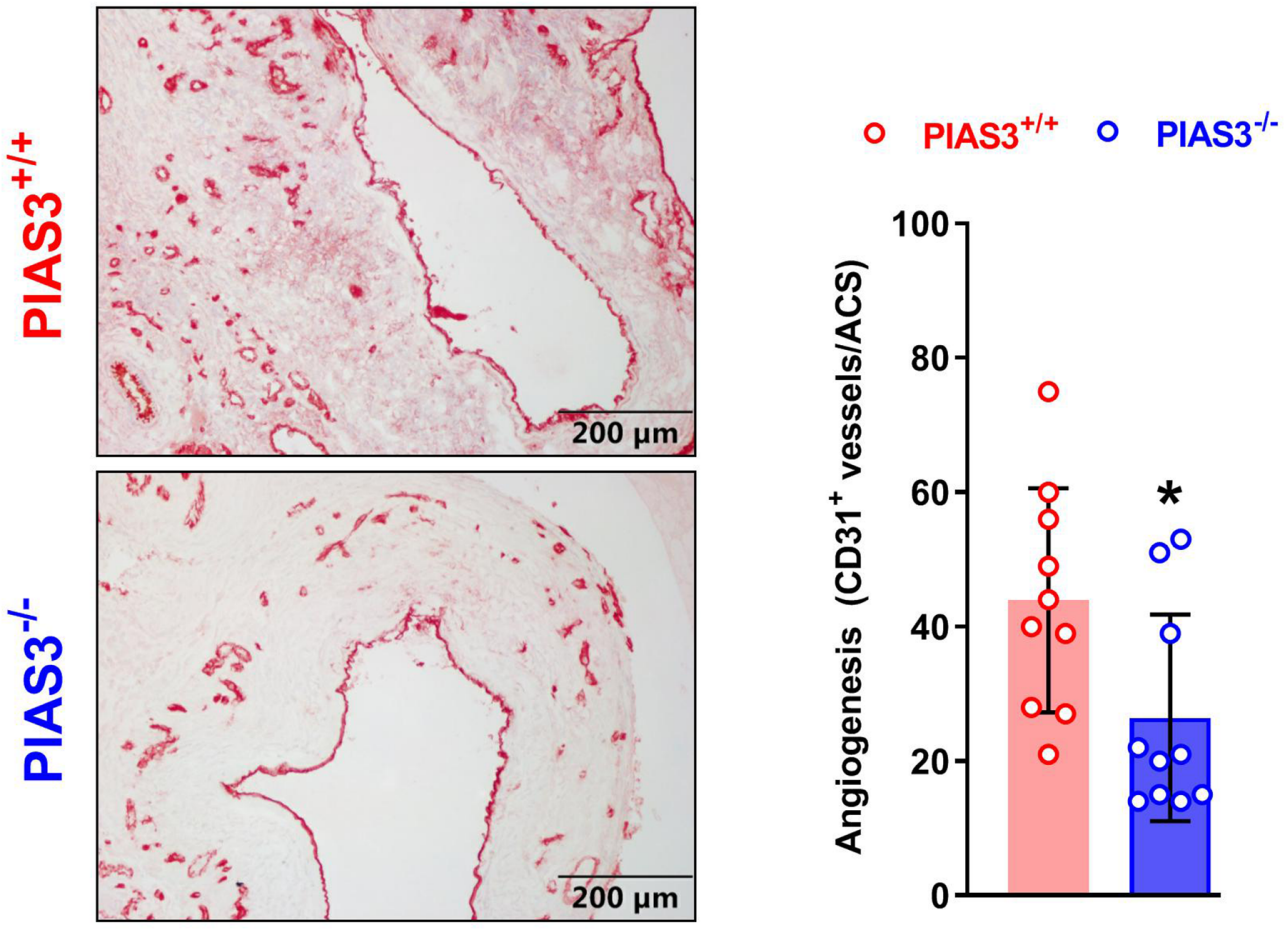
PIAS3 deficiency inhibits mural angiogenesis. Aortic frozen sections were immunostained with an antibody against mouse CD31. Angiogenesis was quantitated as the number of CD31-positive neovessels per aortic cross-section (ACS). *N* = 10 for each group, *p < 0.05 and **p < 0.01 as compared to wild type (PIAS3^+/+^) mice.

Mural angiogenesis is involved in AAA pathogenesis (24–27). We thus determined whether the protective effect of PIAS3 deficiency on AAAs was associated with altered angiogenesis. In immunostaining of CD31, a maker of angiogenesis, the neovessels were significantly less in PIAS3^−/−^ than that in PIAS3^+/+^ mice (Figure 4, left panel), with a 40% reduction in mural neovessels in PIAS3^−/−^ mice (Figure 4, right panel).

## Discussion

Inflammation is implicated in the initiation and evolution of AAAs (28). It leads to aortic medial elastin degradation, the apoptosis and dysfunction of vascular SMC by proteolytic enzymes, free radicals, cytokines, and other inflammatory products (29). JAK2/STAT3 signaling is a main intrinsic pathway for inflammation. It is involved in the creation and sustenance of inflammatory milieus by modulating the expression of cytokines, chemokines, and other mediators (30). In this study, we found that PIAS3 deficiency attenuated experimental AAAs induced by elastase infusion. There were less aneurysmal medial elastin and SMC destruction in PIAS3^−/−^ mice than those in PIAS3^+/+^ mice. Moreover, in comparison with PIAS3^+/+^ mice, aortic leukocyte infiltration, MMP expression and mural angiogenesis were attenuated in PIAS3^−/−^ mice. These results demonstrate that PIAS3 deletion ameliorated experimental AAAs by preserving SMCs, inhibiting aortic leukocyte infiltration, and decreasing angiogenesis.

It has been previously shown that JAK2/STAT3 signaling activity was involved in the pathogenesis of AAAs (13, 31, 32). In human aortic tissues, JAK2/STAT3 expression levels were higher in aneurysmal than those in non-aneurysmal aortas (31). IL-6, a JAK2/STAT3 activator, was elevated in the human aneurysmal as compared to non-aneurysmal aortas (32, 33). However, previously reported influence of STAT3 pathway components on AAAs varied. Disruption of STAT3 signaling in bone marrow–derived cells aggravated whereas myeloid cell-STAT3 deletion had limited effect on experimental AAAs (34). A STAT3 inhibitor attenuated angiotensin II-induced AAA progression in mice through inhibiting vascular inflammation and maintaining autophagy (35), whereas severe angiotensin II-induced AAAs were noted in mice overexpressing SOCS3, another negative regulator of JAK2/STAT3 signaling, in T lymphocytes in association with impaired IL-17 production (34). These discrepancies of STAT3 inhibition/activation concerning AAA progression might be resulted from differential influence on different vascular structural and immune cells. In SMC, the STAT3 inhibitor suppressed inflammation related signaling activation, such as JAK2/STAT3 and NF-kB signaling, which ameliorated AAAs (35). In T cells, overexpression SOCS3 blocked STAT3 signaling activation, thereby significantly impeded Th17 development and decreased interleukin-17 production (34). Reduced IL-17 production is associated with severe vascular inflammation and enhanced susceptibility to aneurysms (36).

We previously found that PIAS3 was negatively associated with the JAK2/STAT3 activation and inflammatory responses in macrophages during atherosclerosis (14). Unexpectedly, the present study showed that PIAS3 deficiency attenuated PPE-induced AAA formation and reduced leukocyte infiltration in experimental AAA lesions. It is possible that chronic activation of STAT3 caused by PIAS3 deficiency may trigger tissue repair responses. In a recent study, SMC-specific SOCS3 deletion was protective against aortic dissection (37). Regionally activating STAT3 in the aorta may initiate host defense mechanisms thereby promoting SMC survival following vascular injury (37). Chronic activation of STAT3 evoked tissue repair responses by altering the phenotype of SMCs, macrophages, and fibroblasts, leading to enhancement of the tensile strength of the aortic wall (37). In addition, mural angiogenesis also contributes to AAA pathogenesis. In present study, PIAS3 deletion decreased aneurysmal mural angiogenesis, which might also result in the attenuation of AAA formation in PIAS3 deficient mice. PIAS3 has been shown to increase the levels of hypoxia-inducible factor (HIF)-1*α*, a critical angiogenic transcription factor, by stabilizing HIF-1 protein (38). HIF-1*α*/vascular endothelial growth factor (VEGF-A) pathway plays a vital role in the development of AAAs (39–43). Thus, angiogenesis inhibition by PIAS3 deficiency may be potentially attributed to low HIF-1 protein stability and thus reduced VEGF-A levels.

In conclusion, the present study demonstrated that genetic PIAS3 deficiency attenuated experimental AAAs in association with reduced medial elastin degradation, SMC depletion, leukocyte infiltration and aortic wall angiogenesis. Due to the limited aneurysmal tissues, the functions of MMPs and STAT3 were not determined in the present study. Further studies on the mechanism of PIAS3 regulating AAA will be conducted in the future.

## Data Availability

The original contributions presented in the study are included in the article/**Supplementary materials**, further inquiries can be directed to the corresponding author/s.
