# Supplementary material for: Genetic deficiency of protein inhibitor of activated STAT3 suppresses experimental abdominal aortic aneurysms
Source: Front Cardiovasc Med. 2023 Mar 15;10:1092555. doi: 10.3389/fcvm.2023.1092555 (PMC10050368; doi:10.3389/fcvm.2023.1092555)
Supplement: Supplementary file 1 [file Table1.docx]

**Supplementary Table S1. Animals and Reagents**

| Animals | | | | | | | | |
| --- | --- | --- | --- | --- | --- | --- | --- | --- |
| Name | Catalog # | | Dilution ratio | | Host | | Source | |
| PIAS3^-/-^ mice | S-KO-06270 | | NA | | NA | | Cyagen Biosciences | |
| Wild type mice | NA | | NA | | NA | | Xi’an Jiaotong University | |
| Reagents and kits | | | | | | | | |
| Name | Catalog # | | Dilution ratio | | Host | | Source | |
| Elastase | E-1250 | | 1:20 | | Porcine pancreas | | Sigma-Aldrich Corp | |
| Proteinase K | P2308 | | 100 µg/mL | | tritirachium album | | Sigma-Aldrich Corp | |
| Streptavidin-peroxidase conjugate | 016-030-084 | | 1:400 | | NA | | Jackson Immuno Research | |
| AEC substrate kit | SK-4200 | | NA | | NA | | Vector Laboratories | |
| BCA Protein Assay Kit | PA115-01 | | NA | | NA | | TianGen Biotech | |
| O.C.T | 4583 | | NA | | NA | | Sakura | |
| Antibodies for IHC/WB | | | | | | | | |
| Target antigen | Clone # | Catalog # | | Dilution ratio | | Host | | Source |
| PIAS3 | NA | 4164 | | 1:1000 | | rabbit | | Cell Signaling Technology |
| β-actin | NA | HC201 | | 1:5000 | | mouse | | TransGen Biotech |
| Mouse SMC alpha actin | NA | NB300-978 | | 1:200 | | Goat | | Novus Biologicals |
| CD68 | FA-11 | 137002 | | 1:200 | | rat | | Biolegend |
| CD4 | GK1.5 | 100402 | | 1:200 | | rat | | Biolegend |
| CD8 | 53-6.7 | 100702 | | 1:200 | | rat | | Biolegend |
| B220 | RA3-6B2 | 103202 | | 1:200 | | rat | | Biolegend |
| CD31 | 390 | 100402 | | 1:200 | | rat | | Biolegend |
| MMP2 | NA | AF1488 | | 1:200 | | Goat | | R&D Systems |
| MMP9 | NA | AF909 | | 1:200 | | Goat | | R&D Systems |
| anti-rat antibody | NA | BA-9400 | | 1:400 | | goat | | Vector Laboratories |
| anti-goat IgG | NA | 705-065-003 | | 1:400 | | donkey | | Jackson Immuno Research |

NA, not applicable.
